# Overexpressed HDAC4 is associated with poor survival and promotes tumor progression in esophageal carcinoma

**DOI:** 10.18632/aging.100980

**Published:** 2016-06-13

**Authors:** Li-Si Zeng, Xian-Zi Yang, Yue-Feng Wen, Shi-Juan Mai, Meng-He Wang, Mei-Yin Zhang, X.F. Steven Zheng, Hui-Yun Wang

**Affiliations:** ^1^ State Key Laboratory of Oncology in South China, Sun Yat-Sen University Cancer Center, Guangzhou, 510060, China; ^2^ Collaborative Innovation Center for Cancer Medicine, Sun Yat-Sen University Cancer Center, Guangzhou, 510060, China; ^3^ Guangdong Esophageal Cancer Institute, Guangzhou, 510060, China; ^4^ Cancer Center of Guangzhou Medical University, Guangzhou, 510095, China; ^5^ Rutgers Cancer Institute of New Jersey, Rutgers University, New Brunswick, NJ 08901, USA

**Keywords:** histone deacetylase 4, esophageal carcinoma, cell proliferation, cell cycle, epithelial-mesenchymal transition

## Abstract

Histone deacetylases (HDACs) mediate histone deacetylation, leading to transcriptional repression, which is involved in many diseases, including age-related tissue degeneration, heart failure and cancer. In this study, we were aimed to investigate the expression, clinical significance and biological function of HDAC4 in esophageal carcinoma (EC). We found that HDAC4 mRNA and protein are overexpressed in esophageal squamous cell carcinoma (ESCC) tissues and cell lines. HDAC4 overexpression is associated with higher tumor grade, advanced clinical stage and poor survival. Mechanistically, HDAC4 promotes proliferation and G1/S cell cycle progression in EC cells by inhibiting cyclin-dependent kinase (CDK) inhibitors p21 and p27 and up-regulating CDK2/4 and CDK-dependent Rb phosphorylation. HDAC4 also enhances ESCC cell migration. Furthermore, HDAC4 positively regulates epithelial-mesenchymal transition (EMT) by increasing the expression of Vimentin and decreasing the expression of E-Cadherin/α-Catenin. Together, our study shows that HDAC4 overexpression is important for the oncogenesis of EC, which may serve as a useful prognostic biomarker and therapeutic target for this malignancy.

## INTRODUCTION

The incidence of esophageal cancer (EC) in developed countries has risen six fold over the past 25 years. Currently the disease is the eighth most common malignant tumor in the world, with an estimated 455,800 new cases and 400,200 deaths annually [1, 2]. Studies show that a total of 50% of EC occurs in China, ranked as the fourth most common cancer and the fourth leading cause of cancer-related death [1, 3]. There are two histological types of EC, esophageal adenocarcinoma (EAC) and esophageal squamous-cell carcinoma (ESCC). In Asian countries including China, ESCC accounts for up to 90% of the EC cases. Although many advances in diagnosis, surgery and adjuvant therapy have been achieved over the past few decades, 5-year survival rate is still only 20 - 30% for EC patients undergone radical surgery without lymph node metastasis (LNM), and 13% for the patients undergone radical surgery with LNM [4-6]. The main reasons for such low survival rate are early LNM and invasion to adjacent tissues and organs, high prevalence of local and regional recurrence, and distant metastasis. The current clinical staging system cannot accurately predict the recurrence, metastasis and prognosis of EC patients. Furthermore, prognostic biomarker is currently unavailable for EC patients. Therefore, identification of novel biomarkers to detect invasion and predict metastasis and prognosis of EC is urgently needed.

The homeostasis of histone acetylation is maintained by two families of enzymes: histone acetyltransferases (HATs) and histone deacetylases (HDACs) [7]. HDACs mediate histone deacetylation and lead to transcriptional repression, which is involved in many diseases, including age-related tissue degeneration, heart failure and cancer [8]. HDACs form a family consisting of 18 enzymes [9] that are classified into four groups (I to IV) according to sequence homologies, and HDAC4, 5, 7 and 9 constitute the class IIa subtype [10]. Recently, HDACs have been reported to be involved in many human cancers, including hematological malignancies, breast cancer, ovarian cancer, cancers of the digestive system, neuroblastoma, prostate cancer, lung cancer, endometrial carcinomas, renal cell carcinomas and bladder cancer [11-18]. HDAC4 is particularly important for cancer development and progression. For example, HDAC4 expression not only is significantly associated with tumor size in malignant thyroid lesions[19] but also promotes tumor growth through suppressing p21 expression in colon cancer [20], glioblastoma [21], ovarian cancer [22], and gastric cancer [23] cells. Therefore, HDAC4 may be a useful biomarker for diagnosis and prognosis of cancer patients or a potential target for anti-cancer therapy.

However, the expression, clinical significance and biological function of HDAC4 in EC remain to be elucidated because HDAC4 only has been reported to be higher in 36 ESCC samples compared with the paired normal esophageal epithelial tissues [24] and there are no more findings on HDAC4 in ESCC. In this study, we first investigated HDAC4 expression in ESCC tissues and its relationship with the prognosis in ESCC patients, and then we further explored the role of HDAC4 in cell proliferation, cell cycle and migration of EC cells and the underlying mechanism.

## RESULTS

### HDAC4 is upregulated in ESCC tissues and EC cell lines

**Figure 1:**
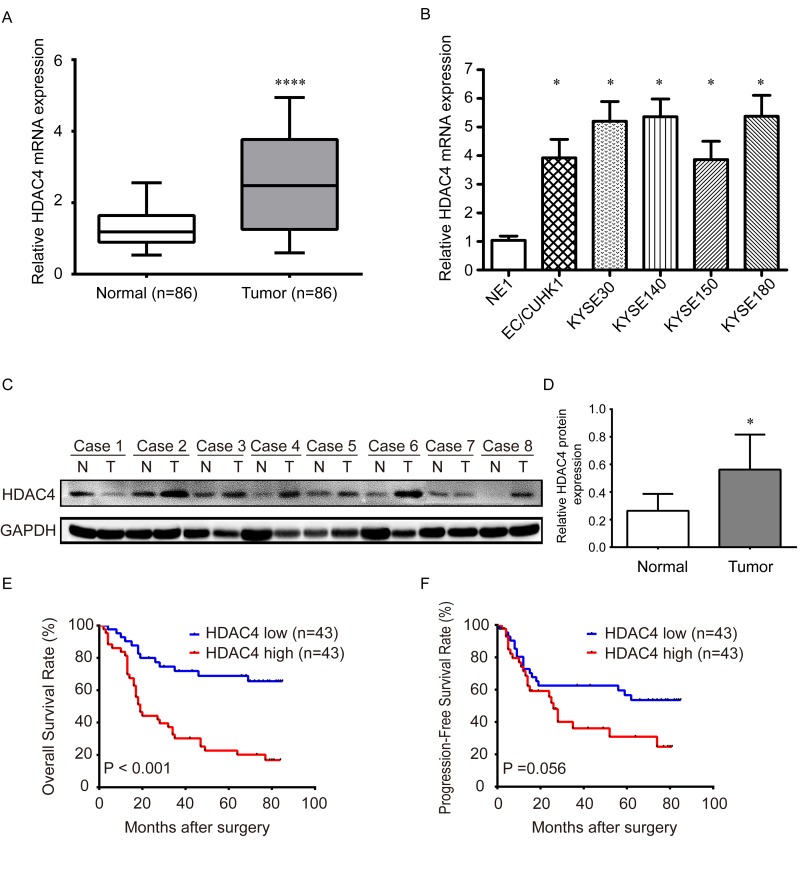
The expression of HDAC4 is upregulated in ESCC tissues and cell lines and associated with survival (**A**) Real-time qRT-PCR analysis shows that expression of HDAC4 mRNA is significantly increased in 86 ESCC tissues compared with the corresponding normal tissues. **** P <0.001; (**B**) The expression of HDAC4 mRNA is significantly increased in EC cell lines, EC/CUHK1, KYSE30, KYSE140, KYSE150 and KYSE180 cell lines, compared with the human normal esophageal cell line NE1. The expression was normalized by GAPDH mRNA. * P <0.05; (**C**) Western blot analysis of HDAC4 protein expression in eight ESCC tissues and paired normal tissues; GAPDH was used as a loading control. (**D**) HDAC4 protein expression in eight ESCC tissues and paired adjacent tissues was quantitated using ImageJ software (Wayne Rashband). (**E**) Kaplan–Meier analysis shows that HDAC4 overexpression is significantly associated with poor overall survival in 86 ESCC cases (P <0.001, log-rank test). (**F**) Kaplan–Meier analysis shows that expression of HDAC4 is marginally significantly associated with poor relapse-free survival in 86 ESCC cases (P =0.056, log-rank test).

The mRNA expression of HDAC4 was analyzed in 86 paired ESCC and adjacent normal tissues by qRT-PCR, which was normalized using GAPDH as an internal control. The results showed that HDAC4 expression was significantly up-regulated in tumor tissues compared with the paired normal tissues (P<0.001) (Fig. 1A). HDAC4 mRNA was also determined in cell lines, which shows significantly higher expression in EC/CUHK1, KYSE30, KYSE140, KYSE150, and KYSE180 EC cell lines than the immortalized human esophageal cell line NE1 (P< 0.05) (Fig. 1B). Western blot indicates that HDAC4 protein is also significantly higher in 8 ESCC tissues than the matched adjacent normal tissues (P< 0.05) (Fig. 1C-D), further confirming that HDAC is overexpressed in ESCC.

### High HDAC4 expression is associated with unfavorable clinical characteristics in ESCC patients

To investigate the clinical significance of HDAC4 overexpression, we analyzed the relationship between HDAC4 mRNA expression and clinical characteristics in ESCC patients. ESCC patients were divided into high and low HDAC4 expression groups using the median expression level as the cut-off point (median, 2.48; range, 0.30 - 6.90). The association between HDAC4 expression level and clinical characteristics was evaluated by Chi-square test. The results show that high HDAC4 expression is significantly correlated with high pathological grade (poor differentiation), T stage, N stage and advanced TNM stage in ESCC patients (Table 1), indicating that HDAC4 overexpression is associated with ESCC progression.

**Table 1:** Correlation between HDAC4 expressions and clinicopathologic characteristics in ESCC

Clinicopathologic characteristics	All cases	HDAC4 mRNA Expression		*P* value
		low	high	
Gender				
female	17	10 (58.8%)	7 (41.2%)	0.417
male	69	33 (47.8%)	36 (52.2%)	
Age (years)				
< 60	44	24 (54.5%)	20 (45.5%)	0.388
≥ 60	42	19 (45.2%)	23 (54.8%)	
Tobacco use				
no	32	19 (59.4%)	13 (40.6%)	0.181
yes	54	24 (44.4%)	30 (55.6%)	
Alcohol use				
no	49	26 (53.1%)	23 (46.9%)	0.514
yes	37	17 (45.9%)	20 (54.1%)	
Tumor location				
upper and middle	65	35 (53.8%)	30 (46.2%)	0.209
lower	21	8 (38.1%)	13 (61.9%)	
Tumor length (cm)				
< 5 cm	57	29 (50.9%)	28 (49.1%)	0.82
≥ 5 cm	29	14 (48.3%)	15 (51.7%)	
Pathological Grade				
I-II	66	38 (57.6%)	28 (42.4%)	**0.011**
III	20	5 (25.0%)	15 (75.0%)	
T stage				
T1-T2	20	16 (80.0%)	4 (20.0%)	**0.004**
T3-T4	66	27 (40.9%)	39 (59.1%)	
N stage				
N0	40	26 (65.0%)	14 (35.0%)	**0.009**
N1-3	46	17 (37.0%)	29 (63.0%)	
TNM stage				
I-II	46	31 (67.4%)	15 (32.6%)	**0.001**
III	40	12 (30.0%)	28 (70.0%)	

NOTE: The bold digit represents *P* value smaller than 0.05. ESCC, esophageal squamous cell carcinoma; TNM, tumor node metastasis.

### HDAC4 is associated with poor prognosis and an independent prognostic factor in ESCC patients

To further explore the prognostic value of HDAC4 in ESCC patients, we analyzed overall survival (OS) and progression-free survival (PFS) of ESCC patients with high or low HDAC4 expression using Kaplan–Meier method and Log-rank test. The 5-year OS and PFS of patients with high HDAC4 expression were only 20.15% and 30.92%, which were considerably shorter than those with HDAC4 low expression (68.75% and 53.59%) (Fig. 1E-1F). Thus, high level of HDAC4 is significantly correlated with poor prognosis of ESCC patients.

To determine whether HDAC4 is an independent prognostic factor in ESCC patients, we analyzed the relationship of HDAC4 expression and various clinicopathological parameters with patient survival using univariate and multivariate Cox proportional hazard models. Univariate analysis indicated that N stage, TNM stage and HDAC4 expression are significant predictors for OS of ESCC patients (Table 2). Moreover, N stage and TNM stage are also significant predictors for PFS in ESCC patients, and HDAC4 is only a marginally significant predictor for PFS in ESCC patients. Multivariate Cox regression analysis demonstrated that HDAC4 is an independent risk predictor for OS (HR: 2.846, 95% CI: 1.456-5.566, P = 0.002) in ESCC patients, not for PFS (Table 2). These results suggest that HDAC4 is a potential biomarker for predicting the overall survival of ESCC patients.

**Table 2:** Univariate and multivariate Cox regression analysis of HDAC4 and survival in patients with ESCC

	Univariate analysis			Multivariate analysis		
Variables	HR	95% CI	*P* value	HR	95% CI	*P* value
**Overall Survival**						
Gender (Female vs male)	1.034	0.515-2.077	0.925			
Age (≥60y vs <60y)	1.558	0.880-2.758	0.129			
Tobacco use (yes vs no)	1.068	0.591-1.931	0.827			
Alcohol use (yes vs no)	1.207	0.681-2.139	0.520			
Tumor location(upper and middle vs lower)	1.494	0.801-2.789	0.207			
Tumor length (≥5cm vs <5cm)	1.503	0.835-2.707	0.174			
Pathological grade (III vs I-II)	1.646	0.881-3.074	0.118			
T stage (III-IV vs I-II)	1.826	0.819-4.072	0.141			
N stage (N1-3 vs N0)	3.056	1.628-5.737	**0.001**	1.443	0.548-3.803	0.458
TNM stage (III vs I-II)	3.264	1.780-5.985	**0.000**	2.359	1.253-4.443	**0.008**
HDAC4 expression (high vs low)	3.740	1.969-7.104	**0.000**	2.846	1.456-5.566	**0.002**
**Progression-Free Survival**						
Gender (Female vs male)	0.549	0.215-1.399	0.209			
Age (≥60y vs <60y)	0.650	0.344-1.228	0.185			
Tobacco use (yes vs no)	1.536	0.769-3.067	0.224			
Alcohol use (yes vs no)	1.018	0.546-1.895	0.956			
Tumor location(upper and middle vs lower)	0.745	0.344-1.614	0.455			
Tumor length (≥5cm vs <5cm)	1.235	0.645-2.364	0.525			
Pathological grade (III vs I-II)	1.273	0.605-2.678	0.508			
T stage (III-IV vs I-II)	1.331	0.614-2.886	0.469			
N stage (N1-3 vs N0)	2.670	1.387-5.141	**0.003**	3.414	1.201-9.708	**0.021**
TNM stage (III vs I-II)	2.026	1.086-3.781	**0.027**	0.703	0.261-1.892	0.486
HDAC4 expression (high vs low)	1.762	0.941-3.297	0.077	1.331	0.682-2.597	0.402

NOTE: The bold digit represents *P* value smaller than 0.05. ESCC, esophageal squamous cell carcinoma; TNM, tumor node metastasis. HR, hazard ratio; CI, confidence interval.

We further stratified ESCC patients by different TNM stage. The results show that stage I-II patients with high level of HDAC4 mRNA have remarkably poorer OS than those with low level (Supplementary Fig. 1A left panel), and stage III patients with high HDAC4 level have marginally significantly shorter OS than those with low level (Supplementary Fig. 1B left panel). It should be noted that the latter is likely to be due to the small sample size. No significant difference is seen between PFS of patients with high and low HDAC4 expression in both stage I-II and stage III subgroups.

Therefore, HDAC4 predicts the survival of ESCC patients independently of clinical stage, particularly in OS, indicating that HDAC4 can provide additional prognostic information for the clinical staging system.

### HDAC4 is important for EC cell proliferation

**Figure 2:**
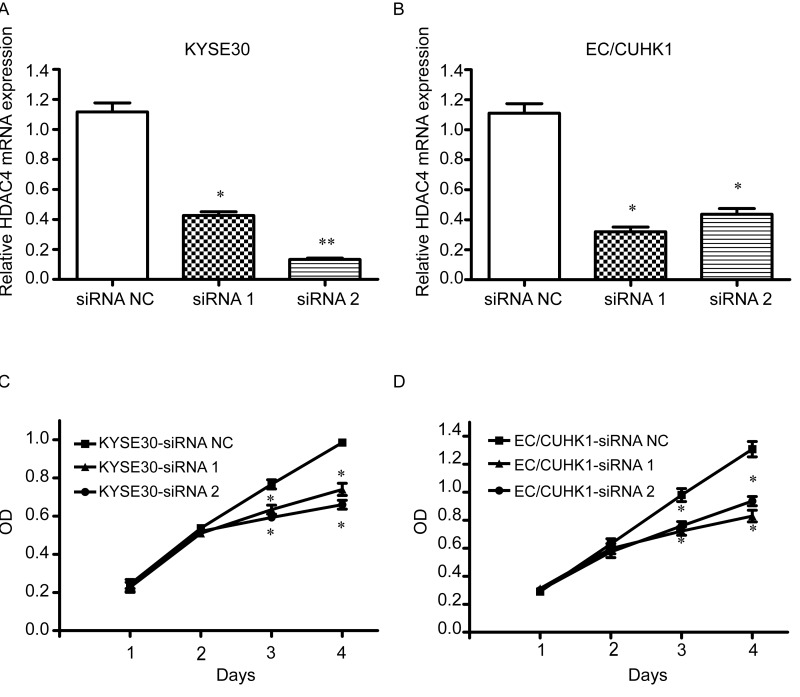
HDAC4 silencing inhibits cell proliferation Real-time qRT-PCR analysis of HDAC4 mRNA expression in (**A**) KYSE30 and (**B**) EC/CUHK1 cell lines transfected with HDAC4 specific siRNAs or a control siRNA. CCK8 assay was then performed to evaluate cell growth of (**C**) KYSE30 and (**D**) EC/CUHK1 cells). Each value represents the mean ± http://S.D.in three independent experiments. * P<0.05; ** P< 0.01. OD, optical density.

**Figure 3:**
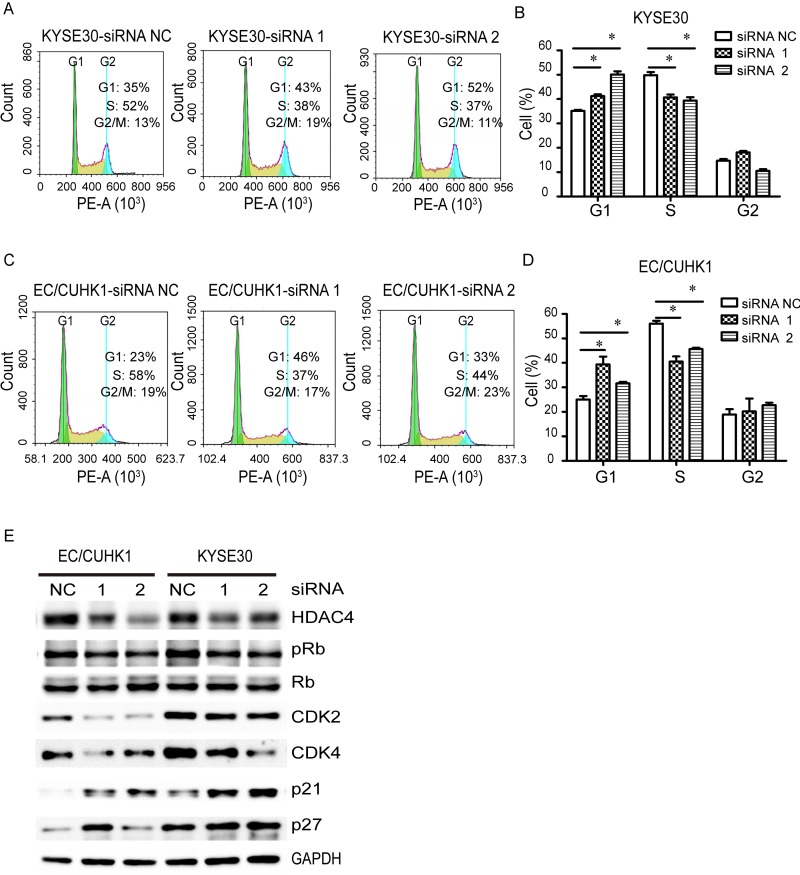
HDAC4 knockdown induces G1/S arrest in EC cells (**A** and **C**) Flow cytometry analysis of indicated EC cells transfected with HDAC4-specific siRNAs or a control siRNA; G1-phase cells are significantly increased while S-phase cells are reduced in HDAC4 knockdown (B) KYSE30 and (D) EC/CUHK1 cells, * P <0.05; (E) HDAC4 down-regulation increases the expression of p21 and p27 proteins while reduces pRb, CDK2 and CDK4 in EC cells.

**Figure 4:**
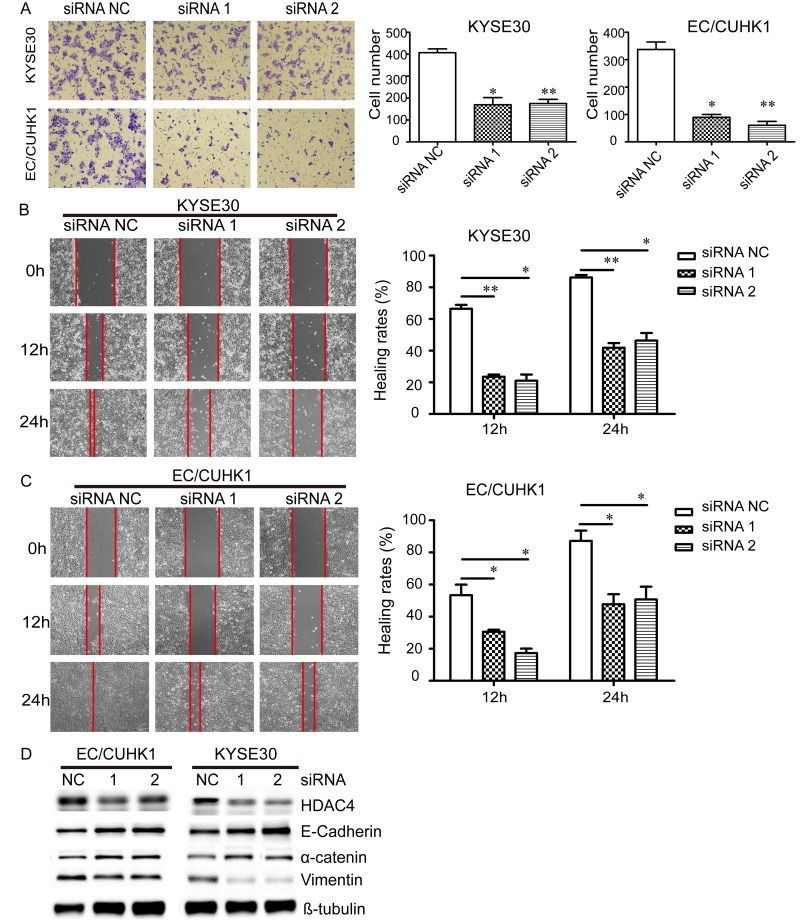
Down-regulation of HDAC4 inhibits the migration of EC cells (**A**) Knockdown of HDAC4 reduces migration of KYSE30 and EC/CUHK1 cells. EC cells were transiently transfected with HDAC4 siRNA or a control siRNA. The number of migrated cells is decreased in cells (x100 magnification) transfected with HDAC4 siRNAs compared with those transfected with a control siRNA. Wound healing assay demonstrates that down-regulation of HDAC4 inhibits cell migration at 12 h and 24 h in (**B**) KYSE30 and (**C**) EC/CUHK1 cells (magnification, x100). (**D**) Western blot analysis indicates that knockdown of HDAC4 increases the expression of E-cadherin and α-catenin, and decreases the expression of Vimentin.

To assess the biological significance of HDAC4 in EC cells, we transfected EC/CUHK1 and KYSE30 cells with two HDAC4 siRNAs and one control (NC) siRNA. The efficiency of siRNA mediated HDAC4 knockdown was evaluated by qRT-PCR and western blot, which show significant reduction of both HDAC4 mRNA and protein (Fig. 2A-2B for mRNA, Fig. 3E and Fig. 4D for protein). The CCK8 cell proliferation assay was further performed. The result shows that HDAC4 siRNAs, not NC siRNA, significantly reduce proliferation of KYSE30 and EC/CUHK1 cells (Fig. 2C and 2D). These results indicate that HDAC4 is important for EC cell proliferation.

### HDAC4 promotes G1/S cell cycle progression in EC cells through downregulating p21/p27 and upregulating CDK2/4 and Rb phosphorylation

Because HDAC4 knockdown inhibits proliferation of EC cells, we asked whether cell cycle is affected by HDAC4 knockdown. Flow cytometry analysis shows that HDAC4 downregulation significantly increases G1 cell population while decreases S cell population in KYSE30 cells (Fig. 3A-B). A similar result is obtained in EC/CUHK1 cells (Fig. 3C-D). These results indicate that HDAC4 knockdown causes G1 cell cycle arrest in both EC cell lines.

The retinoblastoma (Rb) protein is important for G1/S cell cycle transition, which is regulated by two cyclin-dependent kinase (CDK) complexes, CDK4/6-Cyclin D and CDK2-Cyclin E. The CDK inhibitors p21 and p27 oppose the effect of CDK4/6-Cyclin D and CDK2-Cyclin E. We examined whether HDAC4 knockdown affects the level of Rb phosphorylation (pRb), p21/p27 and CDK2/4. Indeed, HDAC4 knockdown in EC/CUHK1 and KYSE30 cells causes a decrease in CDK2/4 and pRb, but an increase in p21 and p27 (Fig.3E). Taken together, our results demonstrate that HDAC4 is important for EC cell cycle progression through CDK2/4, pRb and p21/p27.

### HDAC4 promotes migration of EC cells through enhancing epithelial-mesenchymal transition (EMT)

We also investigated the potential role of HDAC4 in EC cell migration using transwell and wound healing assays. In transwell assay, down-regulation of HDAC4 significantly reduces the number of migrated cell in KYSE30 and EC/CUHK1 cells by approximately 60 % and 80%, respectively (Fig. 4A). In wound healing assay, cell migration rate is markedly decreased in HDAC4 knock-down cells compared with control cells (by approximately 65% and 50% at 12 h and 24 h, respectively) (Fig. 4B-C).

Because HDAC4 knock down inhibits migration of EC cells, we asked if EMT is involved. To this end, Western blot analysis was carried out to determine the expression of E-Cadherin, α-catenin and Vimentin markers for MET. The expression of the epithelial markers E-cadherin and α-catenin are increased, whereas that of the mesenchymal marker Vimentin is reduced in EC/CUHK1 and KYSE30 cells following HDAC4 knockdown (Fig. 4D). These results demonstrate that HDAC4 down-regulation inhibits EMT.

## DISCUSSION

HDACs play an important role in histone modifications essential for normal cell growth, cell cycle progression, differentiation, and development [17]. Unbalanced histone acetylation/deacetylation is often associated with cancer initiation and progression [18]. Many previous studies have been done on the biological functions of HDAC4 in cancer. The clinical significance of HDAC4 in cancers is relatively less well investigated. Halkidou et al report that high level of HDAC4 in nucleus is associated with hormone-resistant in prostate cancer patients [25]. Recently, Gruhn and his colleagues found that high HDAC4 expression is associated with poor survival, high initial leukocyte count, T cell ALL and prednisone poor-response in childhood acute lymphoblastic leukemia [26]. In this study, we find for the first time that HDAC4 mRNA and protein are overexpressed in ESCC tissues compared with matched normal esophageal tissues, and high mRNA level is associated with low differentiation (high pathological grade), lymph node metastasis, large or invasive tumor and advanced TNM stage in ESCC patients. More important, HDAC4 mRNA is an independent predictive factor for OS, suggesting that HDAC4 is a potential biomarker for prognosis in ESCC patients.

HDAC4 has been previously implicated in proliferation, cell cycle progression, apoptosis, angiogenesis and differentiation of cancer cells [17]. For example, inhibition of HDAC4 reduces viability of non-small cell lung cancer [27]. Cadot et al reported that HDAC4 is essential to cell cycle progression in cancer cells independently of p53 status [28]. Zhang et al showed that miR-22 down-regulation is involved in HCC carcinogenesis and progression by enhancing HDAC4 expression [29]. Recently, Vallabhapurapu et al demonstrated that HDAC4 forms a complex with RelB and p52 proteins to promote cell growth by maintaining repressive chromatin at the promoters of proapoptotic genes Bim and BMF in cancer cells, while depletion of HDAC4 or treatment with the HDAC inhibitor TSA results in elevated mRNA levels of both BMF and Bim [30], which demonstrate that HDAC4 may directly influence transcription of specific genes by regulating deacetylation of histones. Furthermore, HDAC4 also promotes development and progression of cancer independently of its chromatin-regulatory function. In this role, it binds to HIF1-α and protects HIF1-α from degradation [31, 32].

In this study, we demonstrate that HDAC4 mRNA is overexpressed in primary ESCCs as well as EC cell lines. HDAC4 knockdown markedly inhibits EC cell proliferation as a result of G1 cell cycle arrest. G1 to S transition is promoted by Rb phosphorylation by CDK2/4, which is opposed by CDK inhibitors p21 and p27 [33, 34]. Our results show that HDAC4 down-regulation leads to inhibition of Rb phosphorylation and CDK2/4 expressions but enhanced expression of p21 and p27 in EC cells. Our results are consistent with the functions of other HDACs in other cancer types. For example, Hrzenjak at al reported that HDAC2 down-regulation results in increased p21 expression [35]; Wilson et al showed that up-regulation of HDAC3 leads to inhibition of p21 expression, and that HDAC3 silencing enhances the expression of p21 in colon cancer cells [36]; Li et al found that HDAC6 down-regulation increases p21 expression and cell cycle arrest in EC9706 cells [37]. Our results further show that down-regulation of HDAC4 elevates p27 expression in EC cell, indicating that p27 is also involved in HDAC4-mediated cell cycle arrest, which has not been previously reported.

Additionally, we show here that HDAC4 down-regulation inhibits EC cell migration due to suppression of EMT pathway (increased E-Cadherin and α-catenin and decreased Vimentin). EMT is a dynamic biological process characterized by loss of polarity, lower adhesiveness, and higher mobility. It is thought to play an important role in cell migration [38, 39]. Our results are consistent with previous reports on other HDACs in cancer cells. In 2013, Li et al reported that down-regulation of HDAC6 reduces the migration of ESCC cells by inhibiting EMT [37]. Hsieh et al found that HDAC6 is required for phthalate-induced cell migration and invasion during EMT in vitro and metastasis into the lungs in nude mice [40]. In conclusions, our study demonstrates for the first time that HDAC4 is overexpressed in ESCC tissues and is an independent predictor for OS in patients with ESCC. Down-regulation of HDAC4 inhibits cell proliferation and G1/S cell cycle progression through reducing CDK2/4-dependent phosphorylation of Rb and elevating CDK inhibitors p21 and p27, as well as cell migration through inhibition of EMT in EC cells. Our findings suggest that HDAC4 promotes the development and progression of EC. It may serve as a prognostic biomarker and therapeutic target for EC.

## MATERIALS AND METHODS

### Tumor samples and cell lines

A total of 28 primary ESCC patients who underwent curative esophagectomy between October 2008 and September 2009 at Cancer Center of Guangzhou Medical University (Guangzhou, Guangdong, China) were enrolled in this study. Another cohort of 58 ESCC patients was recruited at Sun Yat-Sen University Cancer Center, Guangzhou, in 2009. ESCC specimens and paired adjacent normal esophageal epithelium tissues from all 86 patients were collected and snap frozen in liquid nitrogen and stored at −80°C until further use for qRT-PCR and western blot assay. None of the patients received radiotherapy or chemotherapy before surgery. Clinical and pathological information is listed in Table 1. This study was approved by the Research Ethics Committees of Cancer Center of Guangzhou Medical University and Sun Yat-Sen University Cancer Center, and written informed consent was obtained from all patients.

Human cell lines EC/CUHK1, an esophageal carcinoma cell line, and KYSE30, KYSE140, KYSE150, and KYSE180, which are esophageal squamous cell carcinoma cell lines, were cultured at 37°C and 5% CO_2_ in Roswell Park Memorial Institute (RPMI) 1640 medium (Thermo Fisher Scientific, Inc.) with 10% Gibco fetal bovine serum (FBS; Thermo Fisher Scientific, Inc.). NE1 cell line, a normal esophageal cell line, was maintained in a 1:1 mixture of defined keratinocyte serum free medium with growth supplements (Invitrogen) and EpiLife medium with 60 uM Calcium (Invitrogen).

### Total RNA extraction

The total RNA was extracted from tissues and cultured cells using TRIzol reagent (Thermo Fisher Scientific, Inc., Waltham, MA, USA), according to the manufacturer's protocol. The quality and concentration of RNA were determined using a NanoDrop® ND-2000 spectrophotometer (Thermo Fisher Scientific, Inc.).

### Real-time qRT-PCR analysis

Total RNA (400 ng) was employed as template for the reverse transcription (RT) reaction using GoScript Reverse Transcription (RT) System (Promega, USA) according to manufacturer's instructions. Real-time qPCR was performed with 1 μL of RT products (cDNA) and 5μL of GoTaq qPCR Master Mix (Promega, USA) in a 10-μL total volume on a Light Cycler 480 instrument (Roche, USA) according to the manufacturer's protocol. Each sample was analyzed in triplicate wells, and the reactions without cDNA also were included as negative control. The conditions of thermal cycling were as follows: 95°C at 10 min for a hot start, then 45 cycles at 95°C for 15 s, 60°C for 60 s. Glyceraldehyde-3-phosphate dehydrogenase (GAPDH) was used as loading control. The primers were as listed below: HDAC4, forward 5′-GAG AGA CTC ACC CTT CCC G-3′ and reverse 5′-CCG GTCTGC ACC AACCAA G-3′; GAPDH, forward 5′-CTCCTCCT GTTCGACAGTCAGC-3′and reverse 5′-CCCAATAC GACCAAATCCGTT-3′. The 2-^ΔΔCq^ equation was used to represent the relative expression of HDAC4 and Student's t-test was used to analyze the results.

### siRNA transfection

The HDAC4 siRNA1, siRNA2 and negative control (NC) were designed and synthesized by Shanghai GenePharma, Ltd. (Shanghai, China). When the cells reached 60% confluence, 100 nM HDAC4 siRNA1, siRNA2 and NC were transfected into the cells with Invitrogen Lipofectamine® 2000 RNAiMAX reagent (Thermo Fisher Scientific, Inc.), respectively, according to the manufacturer's protocol.

### Cell viability assays (CCK8)

Following transfection for 36 h, EC cells were plated and cultured in 96-well plates (1.0×10^3^ cells/well, six wells per group) for an additional 4 days. The viability of the cells was measured using CCK8 Cell Counting Kit (JingXin Biological Technology, Guangzhou, China). At a specific time every day for 4 days, 10 μl CCK8 was added to each well, followed by additional incubation for 2 h at 37°C. The cells in the 96-well plate were gently agitated for 10 min and the absorbance was measured at 450 nm (SpectraMax® M5 Multi-Mode Microplate Reader; Molecular Devices LLC, Sunnyvale, CA, USA) to calculate the cell viability.

### Cell cycle analysis

EC cells were trypsinized, washed in ice-cold PBS, and fixed in 75% ice-cold ethanol. Cell cycle analysis was conducted by flow cytometry. Briefly, the fixed cells were centrifuged at 1000 r.p.m. and washed twice with ice-cold PBS. Propidium iodide (BD Biosciences, Lexington, KY, USA) staining solution (50 mg/ml, final concentration) were then added and the cells were incubated at 37°C for 30 min in the dark. Cell cycle analysis (20,000 cells per sample) was carried out in a flow cytometer (ACEA NovoCyte^TM^, San Diego, California, USA). Cell cycle analysis software (NovoExpress^TM^, San Diego, California, USA) was used to determine the percentage of cells at each phase of the cell cycle.

### Transwell assay

A Boyden chamber Transwell assay (24-well Transwell plate; BD Biosciences, Franklin Lakes, NJ, USA) was used to measure cell migration, and was performed according to the manufacturer's protocol. EC cells were trypsinized, washed three times with PBS, resuspended (1×10^5^ cells/150 μl) in Serum-free medium, and added to the upper chamber, while 500 μl 10% FBS-containing medium was added to the lower chambers. Following incubation for 24 h, non-migrating cells were removed by a cotton swab, and migrated cells in the filters were immersed in 100% methanol for 15 min at room temperature, and then stained with 0.1% crystal violet (Weijia Biology Science and Technology Co., Ltd) for 30 min at room temperature prior to washing with water. The migrated cells were counted for five random fields with an inverted microscope (BX53; Olympus Corp., Tokyo, Japan). The experiments were conducted in triplicate and repeated three times.

### Wound healing assay

Forty-eight hours after siRNA transfection, a linear scratch wound was performed using a plastic micropipette tip in a confluent monolayer of cells in 6-well plates. Medium without FBS was used in order to inhibit cell proliferation. Then, the cells were washed using PBS in order to wipe off the trashy cells. The remnant EC cells were maintained in RPMI-1640 medium. The distances that the cells moved were observed and recorded.

### Western blotting

EC cells were lysed using the Total Protein Extraction Kit (KeyGEN BioTECH, Jiangsu, China). Following centrifugation at 14,000 x g for 10 min at 4°C, the proteins in the supernatants were quantified using a Bicinchoninic Acid Protein Assay kit (Pierce™; Thermo Fisher Scientific, Inc.) and separated using 10% SDS polyacrylamide gel electrophoresis and transferred to a nitrocellulose membrane (GE Healthcare Life Sciences, Chalfont, UK). The membranes were blocked with 5% nonfat milk in TBST for 2h at room temperature (RT), and incubated with specific primary antibodies at 4°C for 12h, and then with horseradish peroxidase (HRP)-conjugated antibodies for 2h at RT. Detection was performed with electrochemiluminesce (ECL) (Tanon, Shanghai, China). Anti-HDAC4 (Abcam, cat. no. ab12172; 1:1000), Anti-pRb (phospho S780) (ab173289), Anti-Rb (ab85607), anti-p21 (CST, cat. no. mAb #2947; 1:1000), anti-p27 (CST, cat. no. mAb #3686; 1:1000), anti-CDK2 (CST, cat. no. mAb #2546; 1:1000), anti-CDK4 (CST, cat. no. mAb #12790; 1:1000), anti-GAPDH (CST, cat. no. mAb#5174; 1:1000), anti-E-Cadherin (Abcam, cat. no. ab 15148; 1:500), anti-α-catenin (Abcam, cat. no. ab51032; 1:5000), anti-Vimentin (CST, cat. no. mAb #5741; 1:1000), anti-β-tubulin (CST, cat. no. mAb #2148; 1:1000).

### Statistical analysis

Statistical analysis was performed using Student's t test which was used to test the differences between two groups. P value of <0.05 was considered as a statistically significant difference. The association between HDAC4 and clinical characteristics of the patients was analyzed using Student's t-test, χ^2^ test or Fisher's exact test. SPSS version 17.0 software (SPSS, Inc., Chicago, IL, USA) was used for all analysis. P<0.05 was considered to indicate a statistically significant difference.

## SUPPLEMENTARY FIGURE


