# Supplementary material for: Overexpressed HDAC4 is associated with poor survival and promotes tumor progression in esophageal carcinoma
Source: Aging (Albany NY). 2016 Jun 13;8(6):1236–48. doi: 10.18632/aging.100980 (PMC4931829; doi:10.18632/aging.100980)
Supplement: Supplementary file 1 [file aging-08-1236-s001.pdf]

SUPPLEMENTARY FIGURE

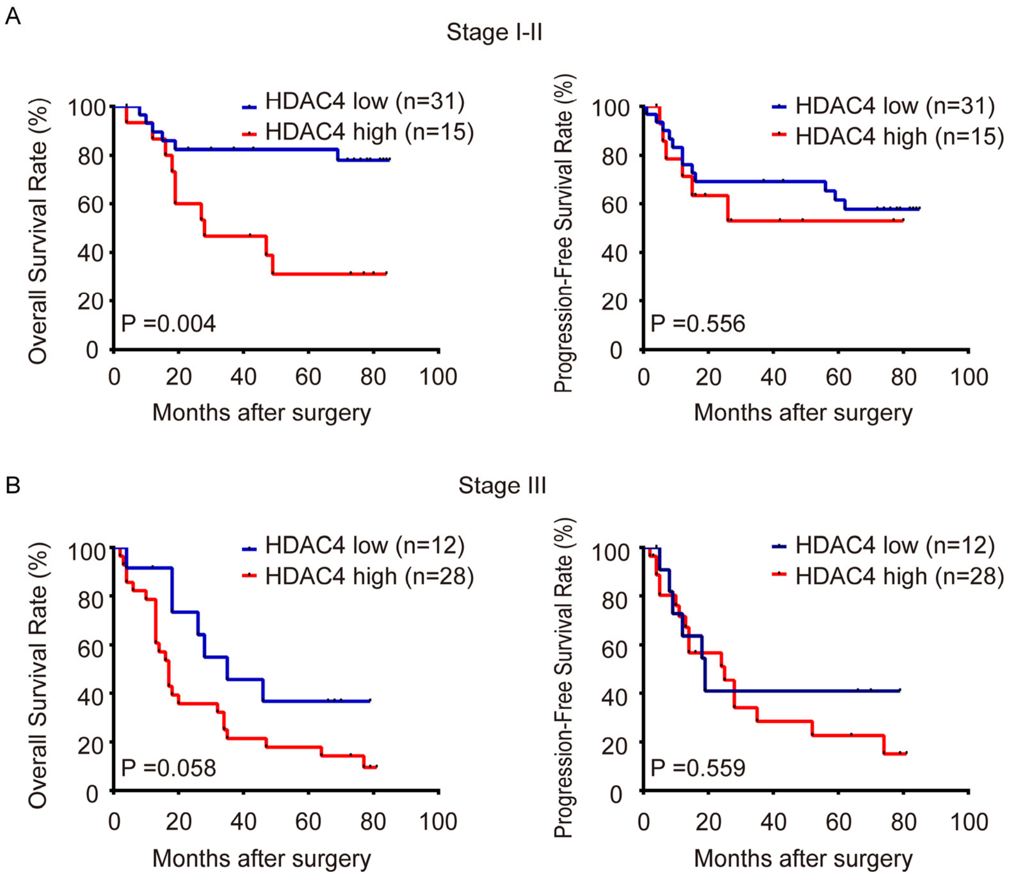

Figure S1. HDAC4 overexpression is associated with poor prognosis for patients with ESCC independent of clinical stage. (**A**) The overall survival (OS) and progression-free survival (PFS) of stage I-II ESCC patients with high or low HDAC4 expression. (**B**) OS and PFS of stage III ESCC patients with high or low HDAC4 expression.
